# Electrohydrodynamic Jet 3D Printed Nerve Guide Conduits (NGCs) for Peripheral Nerve Injury Repair

**DOI:** 10.3390/polym10070753

**Published:** 2018-07-08

**Authors:** Sanjairaj Vijayavenkataraman, Shuo Zhang, Siti Thaharah, Gopu Sriram, Wen Feng Lu, Jerry Ying Hsi Fuh

**Affiliations:** 1 Department of Mechanical Engineering, National University of Singapore (NUS), Singapore 117575, Singapore; zhang.shuo@u.nus.edu (S.Z.); mpestm@nus.edu.sg (S.T.); mpelwf@nus.edu.sg (W.F.L.); jerry.fuh@nus.edu.sg (J.Y.H.F.); 2 Faculty of Dentistry, National University of Singapore, Singapore 119083, Singapore; dengs@nus.edu.sg (G.S.); 3 NUS Research Institute, Suzhou Industry Park, Suzhou 215123, China

**Keywords:** peripheral nerve injury, porous scaffolds, 3D printed scaffolds, tissue engineering, nerve guide conduits, electrohydrodynamic jetting

## Abstract

The prevalence of peripheral nerve injuries resulting in loss of motor function, sensory function, or both, is on the rise. Artificial Nerve Guide Conduits (NGCs) are considered an effective alternative treatment for autologous nerve grafts, which is the current gold-standard for treating peripheral nerve injuries. In this study, Polycaprolactone-based three-dimensional porous NGCs are fabricated using Electrohydrodynamic jet 3D printing (EHD-jetting) for the first time. The main advantage of this technique is that all the scaffold properties, namely fibre diameter, pore size, porosity, and fibre alignment, can be controlled by tuning the process parameters. In addition, EHD-jetting has the advantages of customizability, repeatability, and scalability. Scaffolds with five different pore sizes (125 to 550 μm) and porosities (65 to 88%) are fabricated and the effect of pore size on the mechanical properties is evaluated. In vitro degradation studies are carried out to investigate the degradation profile of the scaffolds and determine the influence of pore size on the degradation rate and mechanical properties at various degradation time points. Scaffolds with a pore size of 125 ± 15 μm meet the requirements of an optimal NGC structure with a porosity greater than 60%, mechanical properties closer to those of the native peripheral nerves, and an optimal degradation rate matching the nerve regeneration rate post-injury. The in vitro neural differentiation studies also corroborate the same results. Cell proliferation was highest in the scaffolds with a pore size of 125 ± 15 μm assessed by the PrestoBlue assay. The Reverse Transcription-Polymerase Chain Reaction (RT-PCR) results involving the three most important genes concerning neural differentiation, namely β3-tubulin, NF-H, and GAP-43, confirm that the scaffolds with a pore size of 125 ± 15 μm have the highest gene expression of all the other pore sizes and also outperform the electrospun Polycaprolactone (PCL) scaffold. The immunocytochemistry results, expressing the two important nerve proteins β3-tubulin and NF200, showed directional alignment of the neurite growth along the fibre direction in EHD-jet 3D printed scaffolds.

## 1. Introduction

Peripheral Nerve Injury (PNI) is a clinical problem caused by the loss of structure and/or function of peripheral nerves because of an accident, trauma, and other causes, which leads to partial or complete loss of sensory, motor, and autonomic functions and neuropathic pain [1]. Though neuroregeneration is possible in PNIs, unlike injuries in the central nervous system, the regeneration is slow and therefore often an incomplete process, especially without any external intervention [2,3]. Hence, surgical intervention is necessary and the current gold standard for treatment is autologous nerve grafting [4]. Autologous tissues such as small nerves, vessels, and muscle are the common sources of nerve autografts [5]. Autologous nerve grafting is considered the gold standard for the repair of PNIs because it reduces the risk of immunological rejection [6] and stimulates a positive therapeutic effect by providing a native tissue microenvironment for nerve regeneration [7]. However, there are many disadvantages associated with nerve autografts, including donor site morbidity, sensory loss, scarring, neuroma formation, and limited supply [8]. Due to the above limitations, alternate treatment methods for the repair of PNIs are actively pursued.

**Scheme 1:**
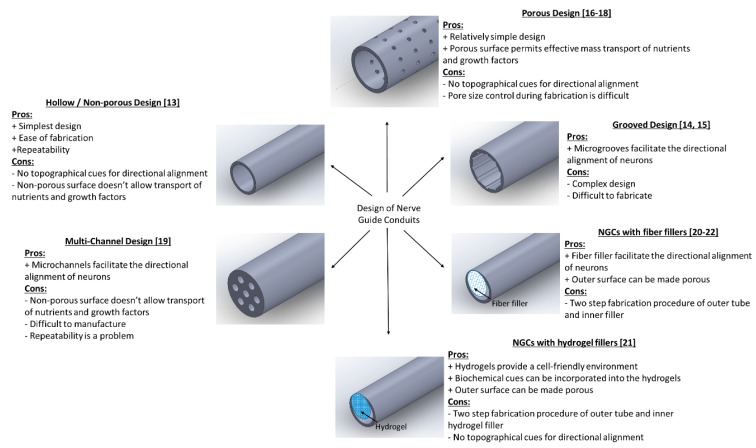
Different designs of Nerve Guide Conduits (NGCs) with their pros and cons.

Neural guide conduits (NGCs) are increasingly being considered as an alternative treatment method to autologous nerve grafting [9]. They are also referred to as artificial nerve conduits or artificial nerve grafts. NGCs have many advantages over nerve autografts, including avoidance of donor site morbidity and unrestricted supply [10,11,12]. There are several designs of NGCs that are reported in the literature. These different designs can be classified under five major groups, namely: (i) Hollow/Non-porous design, (ii) Grooved design, (iii) Porous design, (iv) Multi-channel design, and (v) NGCs with fillers. The hollow or non-porous design is the simplest of all the designs and consists of a hollow tube made of natural or synthetic polymers [13]. The advantages of this design are the ease of fabrication and repeatability. However, the disadvantages outweigh its advantages. Since it is non-porous, the permeability of nutrients and growth factors into and out of the conduit is affected, which in turn will affect the nerve regeneration potential. Another major limitation is the absence of topographical cues for the directional alignment of neurons from the proximal to the distal stump. To address the directional alignment, grooved NGCs are designed where there are microgrooves on the inner surface of the tube to facilitate the directional alignment of neurons [14,15]. However, since this design is also non-porous, the mass transfer across the membrane is still a limitation. The porous design of NGCs came next, which can solve this problem of permeability. Depending on the fabrication method, the porous NGCs can be either fibrous [16,17] or non-fibrous [18]. The porous fibrous design effectively addresses both the limitations of topographical cues for directional alignment (i.e., along the fiber length) and porous surface for mass transport, while non-fibrous design lacks the former advantage. Multi-channel design [19] and NGCs with fillers (fibre fillers or hydrogel fillers) [20,21,22] are two other types of designs that are explored to provide different topographical cues for the alignment of neurons. The various designs of NGCs are pictorially represented in Scheme 1, along with their advantages and disadvantages. Two key properties of an ideal NGC design are the porosity and topographical cues for directional alignment.

Several methods were employed to fabricate NGCs including solvent casting [23], gas foaming [24], phase separation [25], freeze-drying [26], and electrospinning [27,28]. Each process has its own inherent limitations, which are tabulated in Table 1. In addition to these inherent limitations, all the above processes suffer from certain common limitations, including (i) Inability to control the pore size, porosity, and interconnectivity of the scaffolds; (ii) less repeatability; and (iii) no defined multi-layer structure. Of these fabrication methods, electrospinning is the most commonly used technique for the fabrication of NGCs [27,28]. The advantages of using electrospinning are the nanofibrous architecture that provides a larger surface to volume ratio for the cells to attach and grow, the ease of fabrication, and the use of a variety of materials. However, one main disadvantage of electrospinning is that the fibers that are produced are random and highly disordered. Ideal NGC design should have topographical cues for directional alignment as discussed earlier and hence, random fibers are not very desirable. To overcome this challenge, aligned fibers were fabricated using electrospinning by modifying the collector geometry [29,30]. However, there are more challenges yet to be resolved. The pore size, porosity, and interconnectivity of the fabricated scaffold structures cannot be controlled; the process lacks repeatability and customizability; and the process is not scalable.

**Figure 1:**
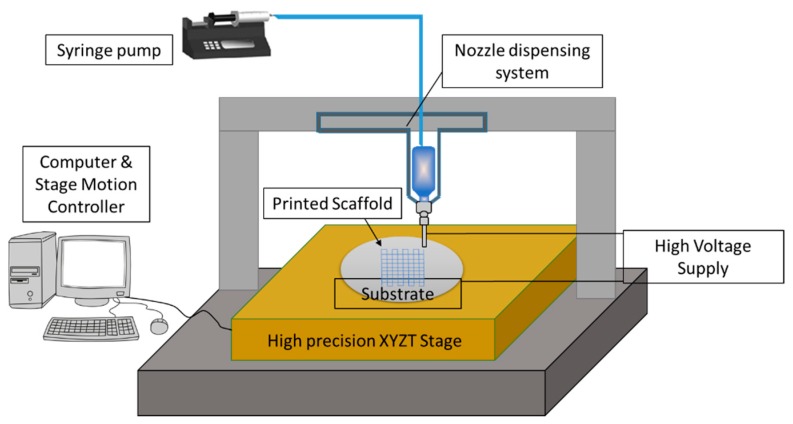
Schematic diagram of the EHD jetting system.

In order to overcome the above limitations of electrospinning, another fabrication technique called 3D printing assisted Electrohydrodynamic jetting (EHD jetting) [31,32,33] is used in this study to fabricate the NGCs. EHD jetting focuses on the control of the EHD jet and patterning and concentrates the fluid flow in the near-nozzle field. The main advantage of this technique is that all the scaffold properties, namely fibre diameter, pore size, porosity, and fibre alignment, can be controlled by tuning the process parameters. The three-dimensional motion stage with precise 3 axes control makes it a layer by layer fabrication process and hence, it is referred to as 3D printing assisted EHD jetting. With this technique, complex geometries can be printed [32] and hence, the process has the advantage of repeatability and customizability. The schematic diagram of the EHD jetting process is given in Figure 1 and the process is briefly described in the Materials and Method section.

Polycaprolactone (PCL) is used as the material in this study to fabricate the NGCs. PCL is a biodegradable, hydrophobic, semi-crystalline polymer and was one of the earliest polymers synthesized. It is an United Stated Food and Drug Administration (FDA) approved material and has proven to be safer for implantation by several in vivo studies [16,17]. PCL is one of the preferred materials for the fabrication of NGCs and there are several published studies on this topic [16,17].

This study focusses on the design and fabrication of porous NGCs by the 3D printing assisted EHD jetting process, with complete control over fibre diameter, pore size, porosity, and fibre alignment. Porous conduits with different pore sizes and porosities are designed. Using PCL as the material, 3D porous scaffolds of different pore sizes are printed (125 to 550 μm). The printed scaffolds are then rolled into tubular conduits and heat-sealed. Mechanical and material characterization of the printed scaffolds and the tubular conduits is conducted. In vitro degradation studies are also performed in order to determine the effect of pore size and porosity on the degradation rate.

## 2. Materials and Methods

### 2.1. Materials

Polycaprolactone (PCL) pellets with an average molecular weight of 80 kDa (Polycaprolactone, Sigma-Aldrich Pte Ltd., Singapore) were used as the solute biomaterial. Glacial acetic acid (>99.7% pure, Sigma-Aldrich Pte Ltd., Singapore) was used as the solvent. All materials were used as purchased.

### 2.2. Preparation of PCL Solution

A PCL concentration of 70% (*w*/*v*, PCL: acetic acid) was used in all the experiments. PCL pellets suspended in acetic acid were ultra-sonicated at 60 °C and 40 kHz for 3 h. It was stirred well every one hour to obtain a homogeneous solution. The solution was left to cool down to room temperature before loading it into the syringe for EHD jetting.

### 2.3. Fabrication of NGCs

An in-house built 3D printing assisted EHD jetting system was used for the fabrication of scaffolds. The schematic of the process is shown in Figure 1. In short, the working principle is based on the balance between the electrostatic force and the combined surface tension and viscoelastic force of the liquid. A high voltage (DC) is applied (typically in the order of 2–3 kV in this experiment) between the nozzle and the substrate, and the surface tension force of the liquid at the nozzle tip is overcome by the electrostatic force between the nozzle and substrate, forcing the solution to come out of the nozzle and be printed on to the substrate. The main components of the system are the high voltage power source, a high precision XYZT stage along with the controller, a syringe pump, and a computer. Software for stage control, connecting tubes, syringes, and needles are other components. A 13 mm internal diameter syringe and 0.5 mm internal diameter needle were used in all the experimental trials. Polished silicon wafers with a diameter of 100 mm were used as substrates. The printed 3D scaffolds were then rolled into tubes with a diameter of 1.2 ± 0.15 mm and the ends were closed by heat-sealing.

### 2.4. Calculation of Theoretical and Experimental Porosity

The theoretical porosity is calculated using the equation below.

П_T_ = 1 − (*V*_solid_/*V*_total_) × 100% (1)

where П_T_—Theoretical porosity, *V*_solid_—the volume of the scaffold with pores or voids (obtained from the modeling software), and *V*_total_—total volume of the scaffold without pores or voids.

The experimental porosity is calculated using the equation below.

П_E_ = 1 − (ρ_s_/ρ_M_) × 100% (2)

where П_E_—Experimental porosity, ρ_s_—the density of the scaffold, and ρ_M_—the density of the material, which is PCL in our case. Additionally,

ρ_s_ = *M*_s_/*V*_s_ (3)

*V*_s_ = *L*_s_ × *W*_s_ × *T*_s_ (4)

where *L*_s_—the length of the fabricated scaffold, *W*_s_—width of the fabricated scaffold, and *T*_s_—the thickness of the fabricated scaffold. The density of PCL (ρ_M_) is 1100 kg/cu·m.

### 2.5. Material Characterization

#### 2.5.1. Scanning Electron Microscope

The EHD jetted scaffolds as printed and the rolled tubular NGCs were sputter-coated with gold (JEOL JFC-1200 Fine Coater, Tokyo, Japan) to render them conductive and visualized using a scanning electron microscope (JEOL JSM-5500). The average fibre diameter and pore size were calculated from the SEM images using image analysis software (ImageJ, National Institute of Health, Bethesda, MD, USA).

#### 2.5.2. Raman Spectroscopy

Raman spectra were recorded on a Horiba Jobin Yvon Modular Raman Spectrometer (Edison, NJ, USA) at a laser excitation wavelength of 514 nm (Stellar Pro Argon-ion laser) for the as-received PCL pellets and EHD jetted scaffolds.

#### 2.5.3. Contact Angle Measurement

Water contact angle represents the degree of hydrophilicity or hydrophobicity of the scaffolds and was measured using the VAC Optima Surface Analysis System (AST Products, Billerica, MA, USA).

### 2.6. Mechanical Testing

Tensile properties of NGCs with five different porosities were determined with a table-top micro-tester (Instron 3345, Norwood, MA, USA) using a load cell of 100 N capacities. Test specimens were tubular NGCs of length 30 mm and diameter 1.2 ± 0.15 mm and were tested at a strain rate of 10 mm/min at ambient conditions. An offset strain of 0.2% was used to determine the yield stress, yield strain, and Young’s Modulus from the stress-strain curves.

### 2.7. Degradation Studies

The NGCs were submerged in individual tubes with 10 mL of Phosphate Buffer Solution (PBS) at a pH of 7.4 with screw caps tightened and maintained at 37 °C in an incubator with a shaker to mimic the physiological conditions. They were removed at selected time points, rinsed thoroughly with deionized water, and blotted dry with clean tissue paper. At each time point, two sets (*n* = 3) of samples were removed. One set of samples was used for subsequent tests in the wet condition and the other set of samples was dried at room temperature for 48 h for subsequent tests. Gravimetric analysis and mechanical testing were performed on the two sets of samples at each time point.

#### 2.7.1. Gravimetric Analysis

Prior to immersion in PBS, the initial weight (*W*_i_) of all the samples was measured. At each time point, samples were removed, rinsed thoroughly with deionized water, and blotted dry with clean tissue paper to remove the excess buffer solution adhering to the surface, dried at room temperature for 48 h, and then weighed (*W*_dry_). The percentage of weight loss was calculated from the equation below.

Weight loss (%) = (*W*_i_ − *W*_dry_)/*W*_i_ × 100% (5)

#### 2.7.2. Mechanical Testing

Mechanical testing was performed on the wet and dry scaffolds to evaluate the effect of degradation on the mechanical properties of the scaffolds. Testing was carried out using the same procedure as outlined in Section 2.7. Test specimens used for degradation studies were rectangular scaffolds with a length of 30 mm and a width of 5 mm and were tested at a strain rate of 10 mm/min at ambient conditions. An offset strain of 0.2% was used to determine the yield stress, yield strain, and Young’s Modulus from the stress-strain curves.

#### 2.7.3. Molecular Weight Determination

The molecular weight changes during each degradation time point were determined by high-performance liquid chromatography using a gel permeation chromatography apparatus (GPC, Agilent 1100, Santa Clara, CA, USA). Scaffolds were dissolved in tetrahydrofuran (THF) at a concentration of 0.1% (1 mg/mL), and filtered through a 0.2 μm inorganic membrane filter prior to the analyses. THF was used as the mobile phase at a flow rate of 1 mL/min. The molecular weights were computed against polystyrene standards.

### 2.8. PC12 Cell Culture

The PC12 cells (a cell line derived from a pheochromocytoma of the rat adrenal medulla) were maintained in Dulbecco’s modified Eagle’s medium (DMEM) high glucose, supplemented with 10% FBS, 5% horse serum, and 1% penicillin/streptomycin. The PC12 cells were incubated at 37 °C in a humidified atmosphere containing 5% CO_2_ and the culture medium was replaced every second day. After reaching 70–80% confluency, the cells were detached by trypsin EDTA and viable cells were counted by a trypan blue assay. Dissociated cells were pelleted by centrifugation at 2000 rpm for 5 min. The pellet was suspended in fresh culture media. Printed PCL scaffolds of varying pore sizes were surface coated with rat tail collagen type I (150 µg/mL solution in distilled water, Invitrogen, Carlsbad, CA, USA) for 1 h at 37 °C in a 24 well plate. Surfaces were rinsed once with distilled water and once with 1× Dulbecco's phosphate-buffered saline (DPBS). PC12 cells were seeded onto the collagen-coated PCL scaffold at a density of 1 × 10^5^ (in 100 µL of culture media) per well and after 4-h incubation, 1.1 mL of culture medium was added to cover the surface. The cell seeded PCL scaffold was left in an incubator at 37 °C in a humidified 5% CO_2_ environment for two days to proliferate. Culture media was changed every two days, with 100 ng/mL NGF added to 1% N_2_ supplemented culture medium, up to six to seven days for neural differentiation of PC12 cells to occur.

### 2.9. Cell Proliferation Using PrestoBlue Assay

The cytotoxicity was evaluated by determining cell viability on day 2 and 7 of incubation with five different pore size scaffolds (125 ± 15, 215 ± 15, 300 ± 15, 400 ± 15, and 550 ± 15 μm). The number of viable cells was determined by estimating their mitochondrial reductase activity using the PrestoBlue (Thermo Fisher Scientific, Waltham, MA, USA) with a Microplate Reader at a wavelength of 570 nm. Briefly, after two and seven days of culture, the scaffolds were moved to new wells and 400 μL of media was transferred to each well. Presto blue reagent was added at a ratio of 10 μL of reagent for every 190 μL of culture media to each well followed by incubation for 45 min. After incubation, 200 μL aliquots were transferred to a 96-well plate from each well, with three replicates per sample. Culture media was set as the blank. Absorbance was measured at a wavelength of 570 nm using a microplate spectrophotometer (Tecan, 200 infinitepro, Männedorf, Switzerland). Experiments were performed in triplicate.

### 2.10. Reverse Transcription PCR (RT-PCR)

To determine the effects of pore sizes on the differentiation of PC12 cells on gene expression, Reverse Transcription-Polymerase Chain Reaction (RT-PCR) was used to detect alterations in the mRNA expressions of genes related to neurite extension: β3-tubulin, Neurofilament Heavy Chain (NF-H), and Growth Associated Protein 43 (GAP-43). Total RNA was isolated from PC12 cells using TRIzol reagent (Thermo Fisher Scientific, Waltham, MA, USA) according to the manufacturer’s recommendations. The purity and quality of the RNA were determined by measuring 260/280 nm ratios. Synthesis of the first-strand cDNA was performed using a Superscript III kit (Thermo Fisher Scientific, Waltham, MA, USA) following the manufacturer’s instructions. The RT-PCR was performed using CFX96™ Real-Time PCR Detection Systems (Bio-Rad Laboratories, Inc., Hercules, CA, USA) and with amplification for 40 cycles. iTaq™ Universal SYBR^®^ Green Supermix (Bio-Rad Laboratories, Inc., Hercules, CA, USA) was used for the PCR reactions. Experiments were performed in triplicate. The primer sequences of genes are shown below (Table 2).

### 2.11. Immunocytochemistry

To observe the neuronal differentiation of PC12 cells for neurite extension on the printed PCL scaffolds, immunostaining of two nerve proteins: neurofilament 200 and β3-tubulin, was carried out. After six days of cell culture, the cell-scaffold constructs were fixed in 10% neutral buffered formalin solution for 15 min and permeabilized with 0.1% Triton-X100 for 10 min. The nonspecific binding was blocked by incubating with 5% BSA for 1 h. Subsequently, the samples were stained with primary anti-body. For NF-H marker, anti-NF200 produced in the rabbit at a dilution of 1:500 at 4 °C overnight, followed by secondary antibody staining using FITC conjugated goat anti-rabbit for 1 h at room temperature. For β3-tubulin, the samples were stained with primary antibody, and β3-tubulin produced in a mouse at a final working concentration of 2 µg/mL at 4 °C overnight, followed by secondary antibody staining using rabbit anti-mouse Alexa Fluor 594 for 1 h. The nuclei were stained with DAPI for 5 min. The immune-stained samples were mounted onto a glass slide and visualized under an Olympus FV1000 laser scanning confocal microscope (Tokyo, Japan).

### 2.12. Statistical Analysis

Experiments were run in triplicate and all measurements were expressed as mean ± SD. A one-way analysis of variance (ANOVA) test was used to determine any significant differences that existed between the mean values of the experimental groups. Differences were considered statistically significant at *p* < 0.05.

## 3. Results

### 3.1. Design of Scaffolds with Different Pore Sizes

**Figure 2:**
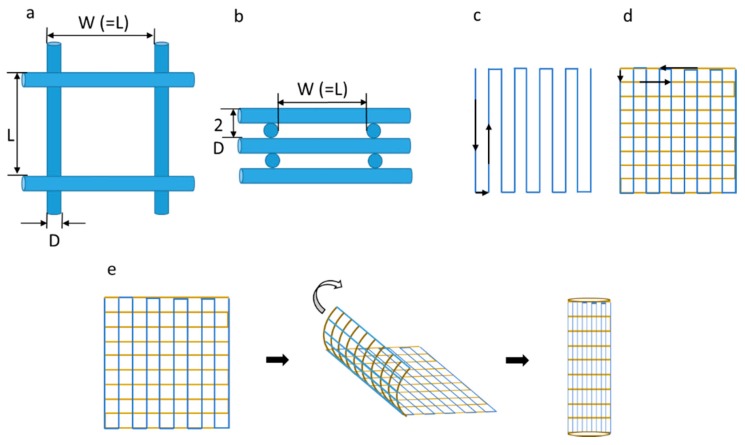
(**a**) Top view of the scaffold (L = W = 125,215,300,400,550 μm); (**b**) Side view of the scaffold; (**c**) Traverse path of the first layer of the scaffold; (**d**) Traverse path of the second layer of the scaffold; (**e**) Rolling of scaffold into NGC.

Scaffolds with five different pore sizes were designed and appropriate program codes were written for the movement of the motion stage as shown in Figure 2a–d. The fibres are printed on the substrate along the vertical direction for the first layer, while they are printed in the lateral direction for the second layer. This pattern was repeated to form a multi-layer scaffold. The printed scaffolds were then rolled into tubular structures with the desired diameter and heat-sealed as shown in Figure 2e.

### 3.2. Effect of Input Voltage, Stage Speed, and Solution Feed Rate on the Scaffold Morphology

**Figure 3:**
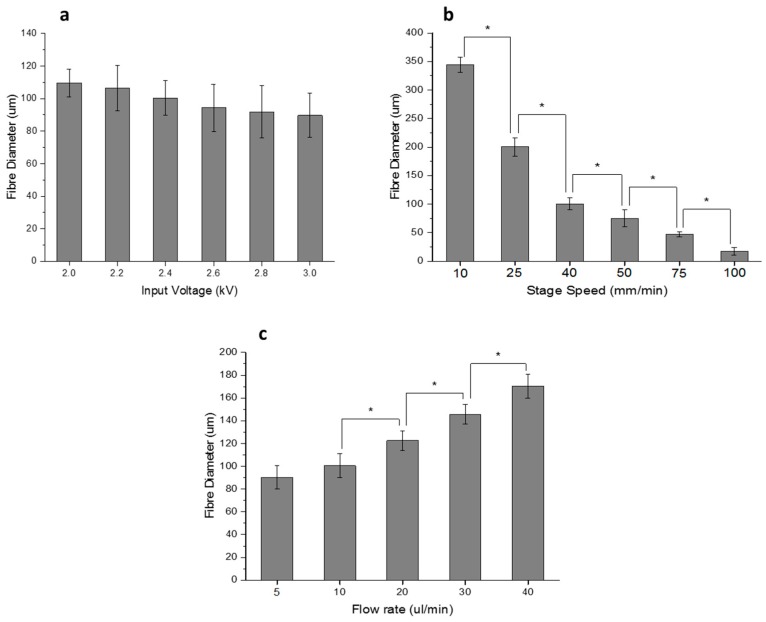
(**a**) Effect of input voltage on fibre diameter (Stage Speed = 40 mm/min, Flow rate = 10 μL/min, nozzle-to-substrate distance = 2 mm); (**b**) Effect of Stage Speed on fibre diameter (Input Voltage = 2.4 kV, Flow rate = 10 μL/min, nozzle-to-substrate distance = 2 mm); (**c**) Effect of Flow rate on fibre diameter (Stage Speed = 40 mm/min, Input Voltage = 2.4 kV, nozzle-to-substrate distance = 2 mm). (Note: * *p* < 0.05).

The important process parameters of EHD jetting that influence the fibre diameter and the scaffold morphology are the input voltage, stage speed, solution feed rate, and nozzle-to-substrate distance. Out of these four parameters, the nozzle-to-substrate distance cannot be varied much as a larger distance will yield random fibres, which is not desirable. Hence, a constant nozzle-to-substrate distance of 2 mm was maintained throughout this study. The other three parameters were varied and the effect on fibre diameter was studied as shown in Figure 3.

The input voltage was varied from 2 up to 3 kV in steps of 0.2 kV. The fibre diameter decreases from 110 to 90 μm as the voltage increases, as shown in Figure 3a. The speed of the high precision stage was varied from 10 up to 100 mm/min. The fibre diameter varies inversely with the stage speed and decreases from 345 to 17 μm as the speed is increased from 10 to 100 mm/min, as shown in Figure 3b. The solution feed rate is varied in the syringe pump from 5 to 40 μL/min. The fibre diameter increases with the feed rate from 90 to 170 μm, as shown in Figure 3c.

### 3.3. Material Characterization

**Figure 4:**
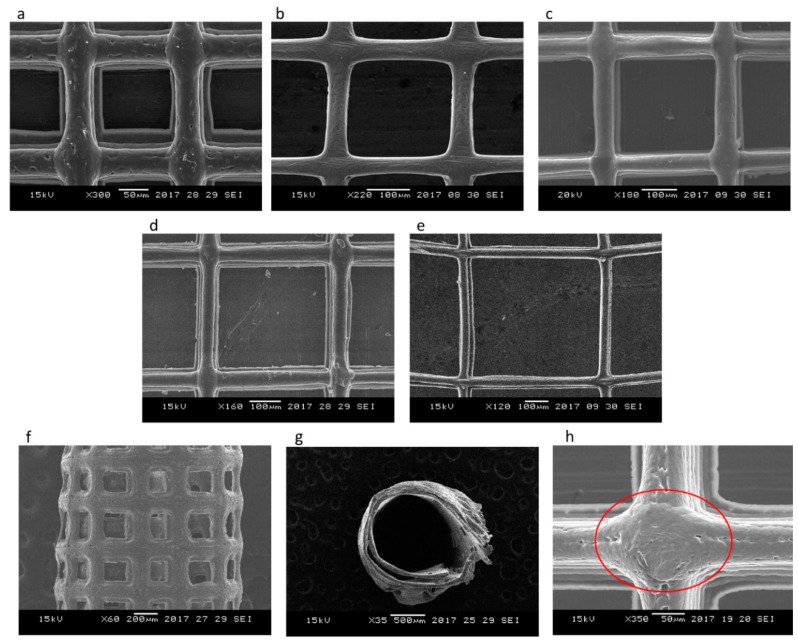
SEM images of PCL scaffolds for Nerve Guide Conduits (NGCs) with different pore sizes fabricated using 3D printing assisted Electrohydrodynamic jetting (Input voltage = 2.4 kV, Stage Speed = 75 mm/min, Flow rate = 10 μL/min, nozzle-to-substrate distance = 2 mm) (**a**) 125 ± 15 μm, (**b**) 215 ± 15 μm, (**c**) 300 ± 15 μm, (**d**) 400 ± 15 μm, (**e**) 550 ± 15 μm, (**f**) front view of the tubular NGC structure with pore size 215 ± 15 μm, (**g**) top view of the tubular NGC structure with pore size 215 ± 15 μm, and (**h**) closer view of the fiber junction.

Figure 4a–e shows the SEM images of PCL scaffolds with five different pore sizes (125 ± 15, 215 ± 15, 300 ± 15, 400 ± 15, and 550 ± 15 μm) and corresponding porosities (65%, 78%, 83%, 86%, and 88%). Figure 4f–g shows the front and top view of the tubular NGC structure with a pore size of 215 ± 15 μm and Figure 4h shows the closer view of the fibre junction. The average fibre diameter from the measurements is 47 ± 5 μm. There are no visible changes in the surface texture or morphology of the scaffolds of different pore sizes as they are made of the same material (PCL). The fibres are uniformly aligned with a defined pore shape, unlike the electrospun fibres/scaffolds.

**Figure 5:**
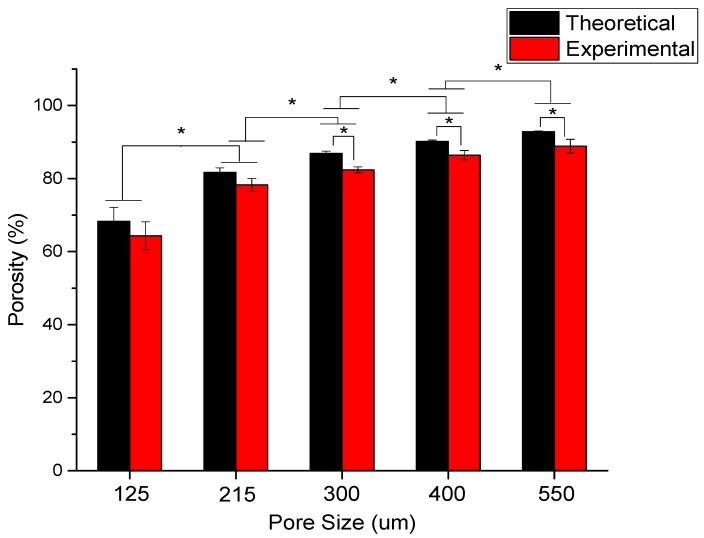
The theoretical and experimental porosity of scaffolds as a function of pore size for different pore sizes (125 ± 15, 215 ± 15, 300 ± 15, 400 ± 15, and 550 ± 15 μm) (* denotes statistical significance (*p* < 0.05)).

The dependence of porosity on pore size is shown in Figure 5 for the scaffolds with five different pore sizes (125 ± 15, 215 ± 15, 300 ± 15, 400 ± 15, and 550 ± 15 μm). The mean experimental porosities of the five groups are 65 ± 4%, 78 ± 2%, 83 ± 1%, 86 ± 2%, and 88 ± 3%, respectively. It can be seen that the experimental porosity value is always less than the theoretical porosity value in all the five groups. The difference between the theoretical and experimental porosity is between 2–5%. Also, it is obvious that the porosity increases with an increasing pore size.

**Figure 6:**
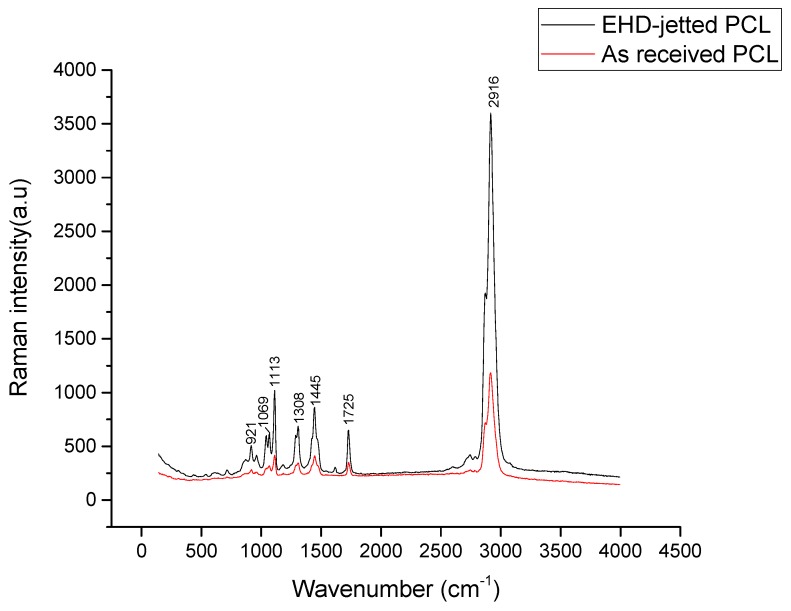
Raman Spectra of as-received PCL pellets and EHD-jetted PCL scaffolds excited with a 514 nm laser line, showing the characteristic peaks of PCL.

The Raman spectrum of the as-received PCL pellets and PCL scaffolds fabricated using 3D printing assisted EHD-jetting is shown in Figure 6, with the band positions and assignments [30] being tabulated in Table 3. The characteristic peaks of PCL, namely the C=O peak at 1725 cm^−1^, CH_2_ asymmetric stretching at 2916 cm^−1^, C–COC crystalline at 1113 cm^−1^, and C–COO crystalline at 921 cm^−1^ are present in both as-received PCL pellets and EHD-jetted scaffolds.

**Figure 7:**
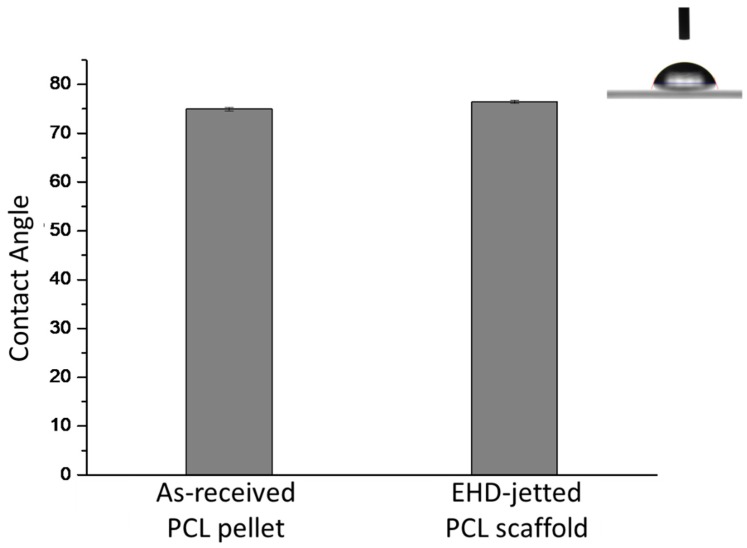
Water contact angle of as-received PCL pellets and EHD-jetted PCL scaffolds (inset image showing the picture of a water droplet on the PCL surface).

The water contact angle results of as-received PCL pellets and EHD-jetted PCL scaffolds are measured to be 75° ± 0.3° and 76.5° ± 0.3°, as shown in Figure 7. There is not much difference in the contact angle and hence the hydrophobicity of PCL had not changed during the fabrication.

### 3.4. Mechanical Testing

**Figure 8:**
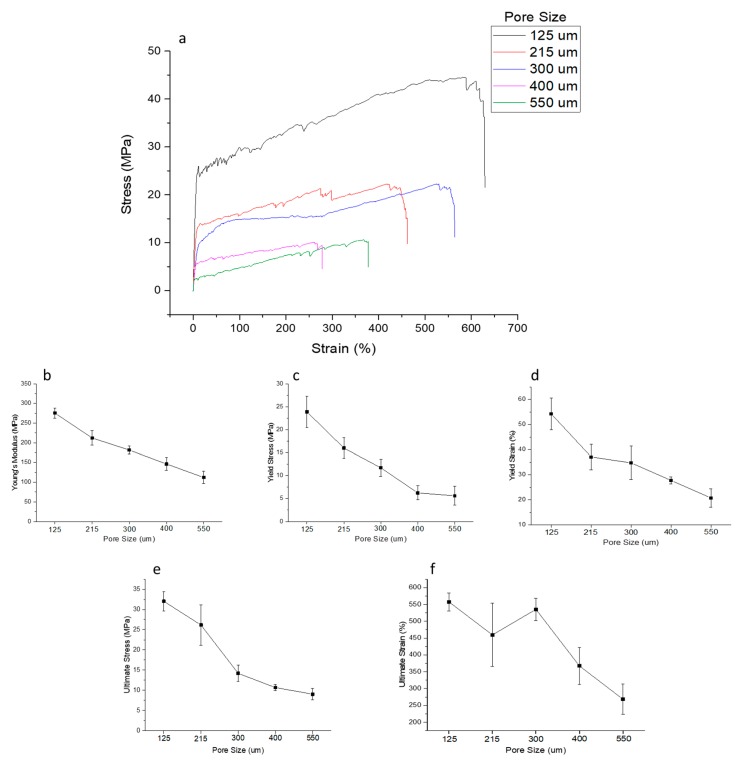
Mechanical properties of NGCs of five different pore sizes (**a**) representative stress-strain curves, (**b**) Young’s Modulus, (**c**) Yield Stress, (**d**) Yield Strain, (**e**) Ultimate Stress, and (**f**) Ultimate Strain.

Stress-strain curves of NGCs with five different porosities are shown in Figure 8a. The Young’s modulus, yield stress, yield strain, ultimate stress, and ultimate strain are also shown in Figure 8b–f. The Young’s modulus of the NGC structure decreases with increasing pore size from 275 ± 13 to 121 ± 16 MPa. Similarly, the yield stress also has a decreasing trend with increasing pore size from 24 ± 3 to 5.6 ± 2 MPa. The ultimate strength of the structure decreases from 32 ± 2.4 to 9 ± 1.4 MPa.

### 3.5. Degradation Studies

The influence of pore size on the degradation rate of PCL scaffolds was assessed using the scaffolds with five different pore sizes (125 ± 15, 215 ± 15, 300 ± 15, 400 ± 15, and 550 ± 15 μm).

**Figure 9:**
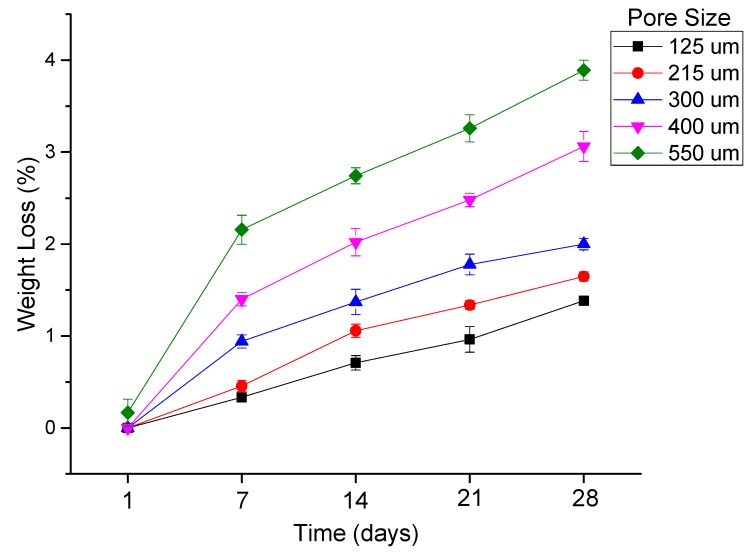
Percentage of weight loss as a function of degradation time of PCL scaffolds with five different pore sizes (125 ± 15, 215 ± 15, 300 ± 15, 400 ± 15, and 550 ± 15 μm).

The percentage of weight loss as a function of degradation time of PCL scaffolds with five different pore sizes (125 ± 15, 215 ± 15, 300 ± 15, 400 ± 15, and 550 ± 15 μm) is shown in Figure 9. Weight loss is directly proportional to pore size, where the smaller the pore size, the lower the degradation rate. The maximum value of weight loss observed was 3.89%, which occurs in the scaffolds with a 550 μm pore size at day 28 and scaffolds with a 125 μm pore size that had a weight loss of 1.38% of their initial weight at day 28.

**Figure 10:**
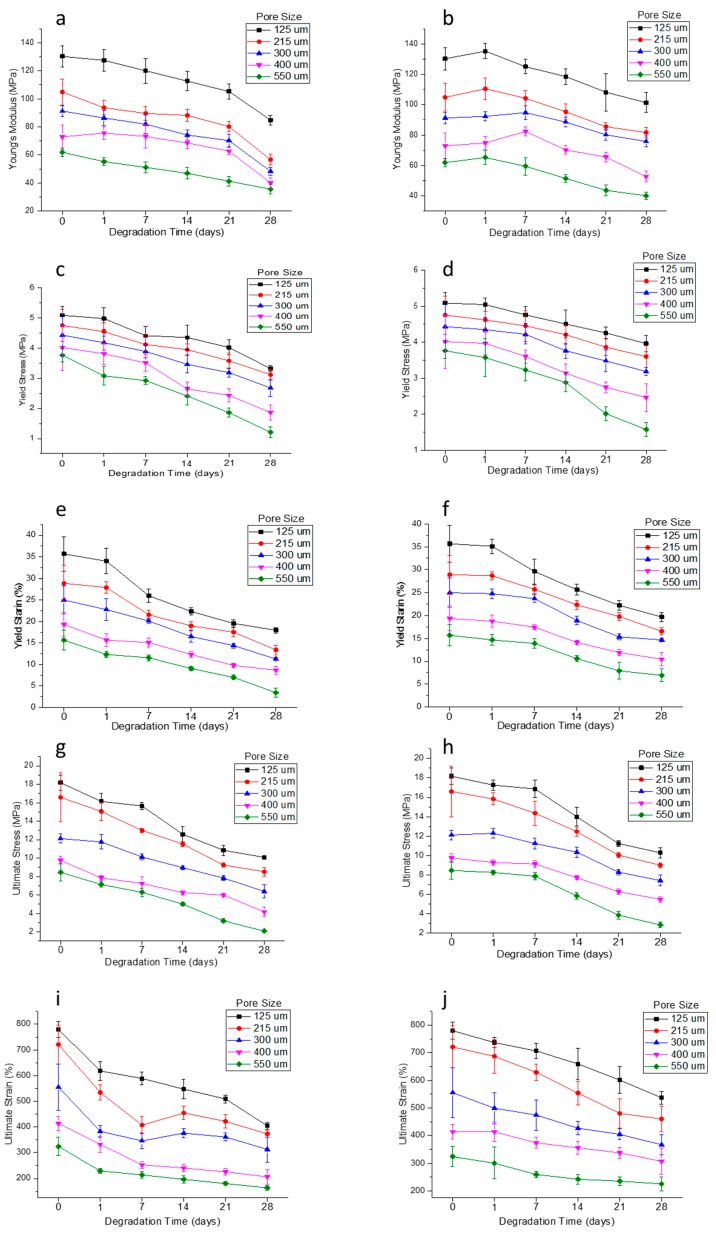
Mechanical properties of PCL scaffolds of five different pore sizes (125 ± 15, 215 ± 15, 300 ± 15, 400 ± 15, and 550 ± 15 μm) in a wet (**a**,**c**,**e**,**g**,**i**) and dry (**b**,**d**,**f**,**h**,**j**) condition (**a**,**b**) Young’s Modulus, (**c**,**d**) Yield Stress, (**e**,**f**) Yield Strain, (**g**,**h**) Ultimate Stress, and (**i**,**j**) Ultimate Strain.

Mechanical testing results at different degradation time points are shown in Figure 10. The results include five mechanical properties, namely Young’s modulus, yield stress, yield strain, ultimate stress, and ultimate strain, for both the wet and dry conditions of the scaffolds, as explained in the materials and methods section. While the mechanical testing reported in Section 3.4 was carried out with the rolled NGC structures, it is to be noted that the degradation studies were performed with as printed scaffolds to determine the effect of pore size on degradation more accurately. It can be seen that the rolled NGC structure has better mechanical properties than the as-printed scaffolds, when comparing Figure 7 and Figure 9. Secondly, the mechanical properties of the scaffolds in their dry state are better than in their wet state, as seen in Figure 9. The difference in the ultimate strain values of the scaffolds in the wet and dry state is very significant (11 to 35%). Thirdly, the mechanical properties were inversely proportional to the pore size and as the pore size increases, the mechanical properties decrease. Also, the percentage decrease of the mechanical properties from day 0 to day 28 was greater in the scaffolds with a greater pore size (550 μm) (~30 to 66%) and was the least in scaffolds with a smaller pore size (125 μm) (~22–45%). Molecular weights of scaffolds were determined using GPC at each of the degradation points. There are no significant changes in the molar mass and dispersity in the samples, even after 28 days.

### 3.6. Calculation of Production Time

In addition to the other advantages that the EHD-jetting process possesses in comparison to other 3D scaffold fabrication methods, calculation of the production time is also possible with this process. The production time of NGCs could be calculated from the following formulae.

Production time = Pre-processing time + Processing time + post-processing time(6)

where,

Pre-processing time = (material preparation time) + (machine set-up time)(7)

Processing time = (length of single fibre) × (number of fibre strands per layer) × (stage speed) × (number of layers)(8)

Pre-processing time remains constant irrespective of the number of samples to be printed. Processing time can be calculated by using Equation (9). If the scaffold length and width, number of layers (thickness), and pore size are known, the number of fibre strands per layer and hence, the processing time, can be calculated. Post-processing includes rolling and heat-sealing in case of NGCs, quality check, and packing time. By multiplying the production time with the hourly rate of man and machine, the production cost for the process can also be calculated.

### 3.7. Cell Culture Studies

PC 12 cells are used in the in vitro cell culture studies of the PCL scaffolds of different pore sizes. A PrestoBlue assay for assessing cell proliferation, Reverse Transcription-Polymerase Chain Reaction (RT-PCR) studies to assess the gene expressions of neural differentiation, and immunocytochemistry studies were conducted.

**Figure 11:**
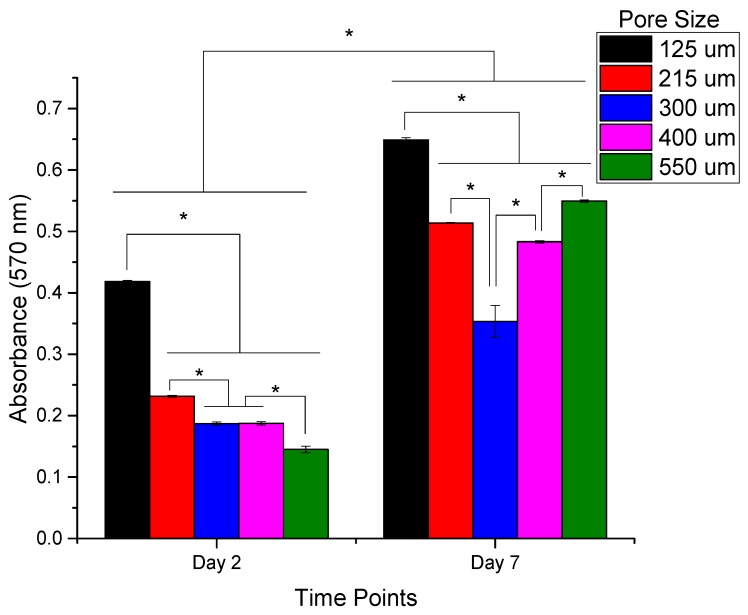
PC 12 Cell Proliferation on PCL scaffolds of five different pore sizes (125 ± 15, 215 ± 15, 300 ± 15, 400 ± 15, and 550 ± 15 μm) on Day 2 and Day 7 of culture days assessed using the PrestoBlue assay (absorbance measured at 570 nm). (*n* = 3) (* denotes statistical significance (*p* < 0.05)).

The cell proliferation and cytotoxicity of the PCL scaffolds with different pore sizes were evaluated using the PrestoBlue assay. The results are shown in Figure 11. It can be seen from the results that the cell proliferation is highest in the scaffolds with a pore size of 125 ± 15 μm on both day 2 and day 7. While on day 2, the cell proliferation decreases gradually with an increase in the pore size from 125 ± 15 to 550 ± 15 μm, on day 7 the trend is non-linear. This non-linear trend can be explained based on the balance between the specific surface area and the ability for cell migration and proliferation [35]. At lower pore sizes, the smaller the pore size, the greater the specific surface area available for cell attachment and proliferation, which can be seen in scaffolds with a pore size of 125 ± 15, 215 ± 15, and 300 ± 15 μm, with decreasing cell proliferation with an increased pore size. However, when the pore size exceeds a certain limit, the effect of specific surface area is overcome by the enhanced cell migration potential of large pores [35].

**Figure 12:**
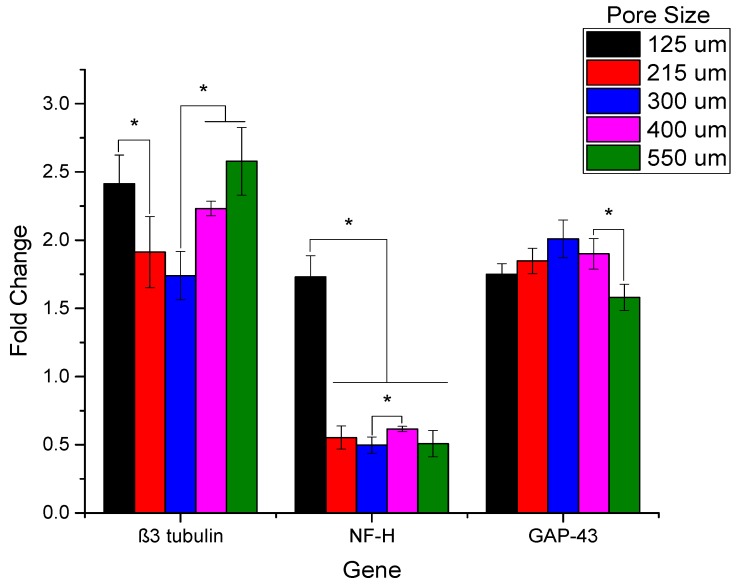
Reverse Transcription PCR analysis of the selected genes in differentiated PC 12 cells grown on PCL scaffold of varying pore sizes (125 ± 15, 215 ± 15, 300 ± 15, 400 ± 15, and 550 ± 15 μm) (*n* =3) (* denotes statistical significance (*p* < 0.05)).

**Figure 13:**
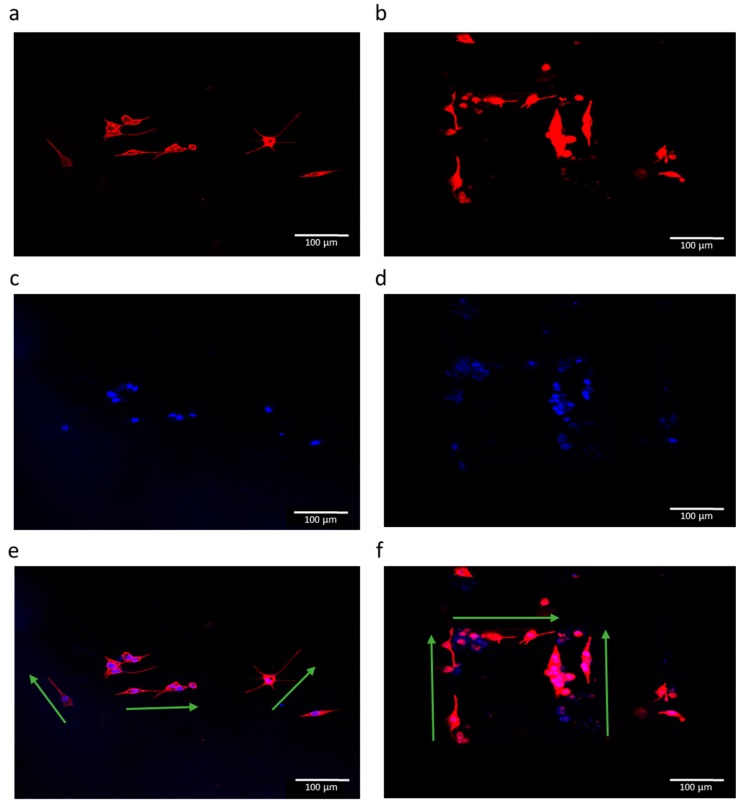
Fluorescence micrographs of PC 12 cells grown on an electrospun PCL scaffold (**a**,**c**,**e**), and EHD-jetted PCL scaffold with pore size 125 ± 15 μm (**b**,**d**,**f**) at culture day 6. Red fluorescence indicates the expression of β3-tubulin, and blue represents the nucleus stained by DAPI. Scale bar: 100 μm (*n* = 3).

**Figure 14:**
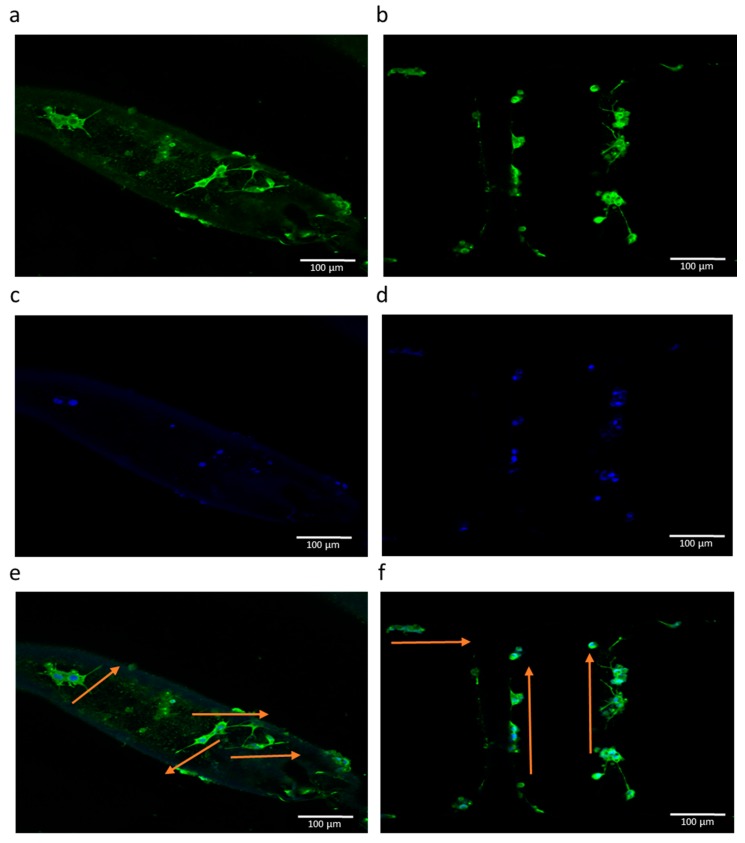
Fluorescence micrographs of PC 12 cells grown on an electrospun PCL scaffold (**a**,**c**,**e**), and EHD-jetted PCL scaffold with pore size 125 ± 15 μm (**b**,**d**,**f**) at culture day 6. Green fluorescence indicates the expression of NF200, and blue represents the nucleus stained by DAPI. Scale bar: 100 μm (*n* = 3).

The results of RT-PCR studies are shown in Figure 12. In addition to the EHD-jetted PCL scaffolds with different pore sizes, an electrospun PCL scaffold (denoted as ES) was also included in this experiment to prove the improved performance of E-jetted scaffolds over electrospun scaffolds. Results are denoted as fold change, where the ES control data was used for normalization. The three most important genes related to neural differentiation, namely β3-tubulin, NF-H, and GAP-43, were considered. GAP-43 is expressed more or less equally in all the samples, indicating that the differentiation happens in all the scaffolds of various pore sizes. The β3-tubulin gene is more highly expressed in scaffolds with pore sizes of 125 ± 15 and 550 ± 15 μm. The NF-H gene expression is higher in scaffolds with a pore size of 125 ± 15 μm. It also outperforms the electrospun PCL scaffold. The immunocytochemistry results of the electrospun PCL scaffold and EHD-jetted PCL scaffold (pore size of 125 ± 15 μm) are shown in Figure 13 and Figure 14. The PC 12 cells expressed the two important nerve proteins β3-tubulin and NF200. Moreover, it can be seen that while the neurite extension was random in the electrospun scaffold, it is aligned along the fibre direction in E-jetted scaffolds (indicated by green arrows in the figure).

## 4. Discussion

NGCs are being considered as an alternative treatment method to autologous tissue grafting in patients with PNS injuries. In this study, we fabricated NGCs using 3D printing assisted EHD-jetting technology, with appropriate scaffold morphology and mechanical properties. Our results demonstrate that the scaffold morphology, which includes the fibre diameter, pore size, porosity, and fibre alignment, can be precisely controlled with EHD-jetting by tuning the process parameters. PCL scaffolds of five different pore sizes (125 ± 15, 215 ± 15, 300 ± 15, 400 ± 15, and 550 ± 15 μm) are fabricated and the effect of pore size on mechanical properties of the NGCs are studied. Our results demonstrate that the mechanical properties decrease as the pore size increases. Degradation of the scaffolds in PBS solution at a pH of 7.4 is also studied to examine the effects of pore size on the degradation rate and the mechanical properties of the scaffold at five time points (Days 1, 7, 14, 21, and 28). Scaffolds with a larger pore size degraded faster than those with a smaller pore size. Mechanical properties deteriorated with the time of degradation.

Biomimetic design is the leading approach in the development of NGCs [36]. Though there were several attempts in fabricating a biomimetic NGC structure using several fabrication methods, the directional control of fibres that serve as the structural cue for neuronal regeneration, repeatability of the process, and scalability are some of the challenges yet to be addressed. The EHD-jetting technique is used as the fabrication method in this study that possesses many unique advantages compared to the other existing scaffold fabrication methods, as outlined in the introduction section. In addition to the advantages of the better control of fibre diameter, fibre alignment, pore size, and ability to fabricate complex biomimetic structures, this technology has the edge in terms of repeatability, customizability, and scalability.

PCL is a biocompatible, biodegradable, semi-crystalline polymer widely used in the field of biomaterials for the fabrication of tissue engineering scaffolds [37,38]. It is an FDA approved material and has proven to be safer for implantation by several in vivo studies [39]. PCL is chosen as the biomaterial in this study due to the many advantages that this polymer has. Firstly, the mechanical properties of PCL are better compared to the other biopolymers. NGCs act as templates for axonal regeneration and should have good mechanical properties [36] and hence, PCL meets the required criterion. Secondly, the rheological properties of PCL are superior to other polymers and hence any polymer processing technology can be used to process them into 3D porous scaffolds with ease [37]. Thirdly, the versatility of PCL that allows modification of its physical, chemical, mechanical, and degradation properties by co-polymerization or blending with other biopolymers due to its exceptional blend-compatibility [40]. While some researchers support the fact that NGCs act as structural templates and hence should possess appreciable mechanical properties, some argue that NGCs should be designed to have mechanical properties that mimic the native nerve [11,36]. Given the exceptional blend-compatibility of PCL, it is easy to tune the properties by adding other biopolymers and varying their relative concentrations to mimic the native nerve properties. Fourthly, PCL is a widely explored biomaterial for polymer-based drug delivery systems due to its compatibility with a wide range of drugs [40] and nerve growth factors can be loaded into PCL fibres in the future for enhancing the repair rate of PNS injuries. Due to all these advantages, PCL is chosen as the scaffold material in this study.

EHD-jetting process parameters play an important role in determining the morphology of the scaffolds. The working principle of this technology is based on the balance between the electrostatic force applied and the combined viscoelastic force and surface tension of the liquid at the nozzle tip [32]. Hence, the four important process parameters are input voltage, solution flow rate, nozzle-to-substrate distance, and speed of the motion stage. In this study, the nozzle-to-substrate distance is kept constant (= 2 mm). Lower values of nozzle-to-substrate distance will result in sparks and higher values will lead to random fibres. We studied the influence of input voltage, the speed of the motion stage, and solution flow rate on the fibre diameter (Figure 3). Fibre diameter decreases with increasing voltage (Figure 3a) due to the increased stretching force. When the voltage is below 2 kV, the fibre formation discontinues as the applied electrostatic force is not enough to produce the Rayleigh-plateau instability and could not overcome the surface tension and viscoelastic force of the liquid. When the voltage is increased beyond 3 kV, given that the nozzle-to-substrate distance is 2 mm, sparks are produced. Stage speed has a greater influence on fibre diameter (Figure 3b) and they are inversely proportional to each other. Fibre diameter decreases remarkably from 345 to 17 μm when the speed is increased from 10 to 100 mm/min. At higher speeds, the volume of liquid dispensed at a particular point on the substrate is reduced and hence, the fibre diameter decreases. The additional pulling force of the moving stage on the dispensed jet also contributes to the decreasing fibre diameter. When the solution feed rate is increased, the fibre diameter increases due to the increased volume of liquid dispensed as expected. For all the three parameters, the trend agrees with the earlier studies published on this subject [31,32,41,42]. From these studies, the capability of EHD-jetting to fabricate scaffolds with different fibre diameters and pore sizes at the desired speed (that influences the production time) is proven.

With the understanding of the influence of the interplay between the various process parameters on the scaffold morphology, PCL scaffolds of five different pore sizes (125 ± 15, 215 ± 15, 300 ± 15, 400 ± 15, and 550 ± 15 μm) are fabricated to study the effect of varying pore size on the mechanical properties of the scaffolds. Figure 4 shows the SEM images of these scaffolds. A highly aligned straight fibre can be seen in all the images with different pore sizes. The rolled NGC structure (Figure 4f,g) mimics the dimensions of the native peripheral nerve with a diameter of 1.2 mm, wall thickness of ~200 μm, length of 1–3 cm, and porosity greater than 60% [43], and with highly aligned fibres that serve as the directional cue for directing the regenerated neurons. One unique advantage of EHD-jetting compared to the other methods such as solvent casting, freeze drying, or melt moulding, is that the fibre diameter (wall thickness) and pore size can be controlled individually. In the other processes, these two are interdependent. In other words, pore size is increased by decreasing the wall thickness and vice versa [44,45,46]. Hence, with E-jetting, it is possible to fabricate scaffolds of different pore sizes and porosities but with the same fibre diameter. Theoretical and experimental porosities as a function of pore size are shown in Figure 5. It can be seen that the experimental porosity is always lower than the theoretical porosity values. This is due to the morphological differences between the design and the fabricated scaffolds, as shown in Figure 4h. While the fibres in the adjacent layers of the scaffolds are perfectly tangential with each other in the design (Figure 2a,b), the fibres merge at the junctions in the EHD-jetted scaffolds (as indicated by the red circle in Figure 4h), thus reducing the porosity value by 2–5%. The mean experimental porosities of the five groups are 65 ± 4%, 78 ± 2%, 83 ± 1%, 86 ± 2%, and 88 ± 3%, respectively.

Raman spectroscopy is done on both as-purchased PCL pellets and EHD-jetted scaffolds to investigate if there are any differences in the material structure or composition. The characteristic peaks of PCL, namely the C=O peak at 1725 cm^−1^, CH_2_ asymmetric stretching at 2916 cm^−1^, C–COC crystalline at 1113 cm^−1^, and C–COO crystalline at 921 cm^−1^, are present in both as-received PCL pellets and EHD-jetted scaffolds [34], demonstrating that the material has not undergone any alterations during the fabrication process. The contact angle measurement on as-received PCL pellets and EHD-jetted scaffolds reveals that the hydrophobicity of PCL is also not affected due to the fabrication method.

The influence of pore size on the mechanical properties of the NGC structure is studied (Figure 8). With increasing pore size and porosity, the mechanical properties, namely Young’s modulus, yield stress, yield strain, ultimate stress, and ultimate strain, decrease. The results are in line with previous published studies [47,48,49]. Higher porosity and larger pore size scaffolds facilitate the better mass transfer of nutrients, growth factors, and oxygen, and also enable more cell ingrowth [49]. However, the mechanical properties are compromised as the pore size and porosity are increased. There is an optimal range of porosity value according to the intended application where there is a balance between the nutrient exchange facilitation and sufficient mechanical properties to maintain structural integrity until new tissue regeneration. For NGCs, the optimal porosity range reported from the previous studies is 60–80% [43] and scaffolds with porosities greater than 80% were reported to cause guide mechanical instability [50]. EHD-jetted scaffolds with pore sizes 125 ± 15 and 215 ± 15 μm have the porosity within this range. Also, it can be seen from the mechanical testing results that the ultimate tensile strength of the scaffolds with pore sizes of 125 ± 15 and 215 ± 15 μm falls in a range closer to the native peripheral nerve (6.5 to 11.7 MPa) [51,52] throughout the degradation cycle of 28 days, with an optimal pore size and porosity.

In vitro degradation studies are performed to evaluate the effect of pore size and porosity of the scaffolds on the degradation process. It is highly recommended that the influence of pore size and porosity on the degradation rate has to be taken into account, in addition to designing porous scaffolds with an optimum pore size range for a given application [45]. While there are several studies on the influence of porosity on the degradation rate, the effect of pore size on the process of degradation is not well-established [45]. The main reason for this is the inter-dependability of wall thickness and the pore size, as stated earlier, which is an inherent limitation of the fabrication process [44,45,46]. This study clearly eliminates the problem as a constant fibre diameter is maintained for all the scaffolds with five different pore sizes. Our results (Figure 9 and Figure 10) indicate that scaffolds with a larger pore size degrade faster than those with smaller pore sizes. Scaffolds with a pore size of 550 μm degraded faster with a weight loss of 3.89%, while in scaffolds with a pore size of 125 μm, the weight loss is only 1.38% after 28 days. The trend agrees with the previous studies published [45,46]. Scaffolds with smaller pore sizes degrade slowly due to the slower rate of hydrolysis and scaffolds with larger pore sizes degrade faster due to auto-catalyzed degradation as larger pore sizes allow more PBS solution to be contained per unit volume of scaffolds [45,46,53]. Mechanical properties are also studied at various time points during the scaffold degradation for 28 days. Previous studies on in vitro degradation of electrospun PCL scaffolds reported a reduction in the mechanical properties to nearly half of the initial condition after 28 days [54,55]. In our study, the percentage reduction of mechanical properties ranged from 22 to 66% based on the pore size. The mechanical properties were inversely proportional to the pore size and as the pore size increases, the mechanical properties decrease. The percentage decrease of the mechanical properties from day 0 to day 28 was greater in the scaffolds with a greater pore size (550 μm) (~30 to 66%) and was the least in scaffolds with a smaller pore size (125 μm) (~22–45%). It is also known from the previous studies that the in vivo degradation of PCL scaffolds is much faster than in vitro degradation [55]. The average rate of peripheral nerve regeneration is 1 mm/day [56,57] and PNS injury with a 3 cm gap takes approximately 30 days to regenerate. Therefore, the NGCs are expected to be structurally intact for at least 30 days for long gap injuries and the EHD-jetted PCL based NGCs fabricated in this study are suitable for this application. Given the fact that the peripheral nerve regeneration rate after injury may vary from a few days to over three years [56,57] depending on the site and severity of injury, PCL is a good choice of material that has a slower degradation rate. In addition, due to its exceptional blend capability, the degradation rate of PCL can be tuned according to the regeneration rate of the target peripheral nerve by the addition of other biopolymers to PCL.

GPC did not show significant changes in the molar mass and dispersity in the samples, even after 28 days. This is due to the high initial molecular weight (80 kDa) and long degradation time (>1 year) of PCL. Given the small magnitude of the observed weight loss (<4%) and no significant molar mass changes throughout the degradation period, together with the significant changes of mechanical properties, further detailed morphological studies including DSC are needed to investigate the degradation behavior in depth.

In addition to the capability of fabricating biomimetic NGCs with an appropriate pore size, mechanical properties, and degradation rate, EHD-jetting also possesses repeatability and scalability. The production time of scaffolds can be almost accurately predicted with the formulae given in Section 3.6 for better planning of production and cost estimation.

The in vitro neural differentiation studies performed with the different scaffolds suggest that the EHD-jetted scaffold with a pore size of 125 ± 15 μm is the one best-suited for NGC. The highest proliferation was seen in the scaffolds with a pore size of 125 ± 15 μm, as evidenced by the PrestoBlue assay results (Figure 11). The results of RT-PCR studies (Figure 12) involving the three most important genes concerning neural differentiation, namely β3-tubulin, NF-H, and GAP-43, confirm that the scaffolds with a pore size of 125 ± 15 μm has the highest gene expression of all the other pore sizes and also outperforms the electrospun PCL scaffold, which is a commonly used method for the fabrication of NGCs. While the GAP-43 gene is expressed equally on all the scaffolds, there are significant differences in the expression of β3-tubulin and NF-H. Scaffolds with a pore size of 125 ± 15 μm have the highest expression of both β3-tubulin and NF-H. Although 550 ± 15 μm also has a high expression of the β3-tubulin gene comparable to the former, given the biomimetic mechanical properties and appropriate degradation rate, scaffolds with a pore size of 125 ± 15 μm are preferred. The immunocytochemistry results concerning the two most important structural nerve proteins, namely β3-tubulin and NF200 (shown in Figure 13 and Figure 14, respectively), also suggest that the EHD-jetted scaffolds with a pore size of 125 ± 15 μm are the best compared to the electrospun scaffold, with the directional alignment of the neurite growth along the fibre direction.

## 5. Conclusions

EHD-jetting technology offers several advantages over the other existing tissue engineering porous scaffold fabrication methods. EHD-jetting is used for the fabrication of NGCs for the first time in this study. The study on the effect of various process parameters on the scaffold morphology demonstrates the flexibility of this technology to fabricate scaffolds with a complex geometry, fibre diameter, and pore size with ease. PCL is chosen as the material due to its biocompatibility, biodegradability, manufacturability, and exceptional blend-compatibility. The pore size of the scaffolds plays an important role in determining the mechanical properties of the structure. Our results indicate that scaffolds with a larger pore size and high porosity have weaker mechanical properties and the degradation rate of the scaffolds increases with increasing pore size and porosity. EHD-jetted scaffolds with a pore size of 125 ± 15 μm are found to possess the desirable properties of an ideal NGC structure with a porosity greater than 60%, mechanical properties closer to those of the native peripheral nerves, and an optimal degradation rate matching the nerve regeneration rate post-injury. The in vitro neural differentiation studies also prove that the scaffolds with a pore size of 125 ± 15 μm support the highest differentiation, as evidenced by the RT-PCR and immunocytochemistry studies.

## Figures and Tables

**Table 1:** Limitations of current NGC Fabrication Technologies.

Fabrication Technologies	Limitations
Solvent Casting	Highly toxic solvent, poor interconnectivity, irregularly shaped pores, low porosity (<50%)
Gas Foaming	Poor interconnectivity, external surface is non-porous
Phase Separation	Limited only to specific polymers
Freeze Drying	Irregularly shaped pores
Melt moulding	Presence of residual porogen particles, high processing temperatures
Electrospinning	Fibres produced are random and highly disordered; lacks repeatability and customizability

**Table 2:** Primer sequences of genes used for RT-PCR.

Gene	Primers	Length
GAPDH	**Forward 5′** CGTGGAGTCTACTGGCGTCTTC **3′** **Reverse 5′**GGGAGTTGTCATATTTCTCGTGGTT **3′**	22 25
β3 tubulin	**Forward 5′**CAGATGCTGGCCATTCAGAGTAAG **3′** **Reverse 5′** TGTTGCCGATGAAGGTGGAC **3′**	24 20
Neurofilament-Heavy chain	**Forward 5′**AAGGAAACCGTCATTGTAGAGGAA **3′** **Reverse 5′** GGAGACGTAGTTGCTGCTTCTT **3′**	24 22
GAP-43	**Forward 5′** CCGACAGGATGAGGGTAAAG **3′** **Reverse 5′** GCAGGAGAGACAGGGTTC **3′**	20 18

**Table 3:** Observed significant Raman bands (cm^−1^) and their assignments for EHD-jetted PCL Scaffolds excited with a 514 nm laser line [34].

Raman Bands (cm^−1^)	Assignments
921	ν(C–COO); crystalline
1069	ν(COC); crystalline
1113	ν(COC); crystalline
1308	ω(CH2); crystalline & amorphous
1445	δ(CH2); crystalline
1725	ν(C=O); crystalline
2916	Antisymmetric C–H stretching ν(CH_2_)_asym_
